# TCR Signal Strength Alters T–DC Activation and Interaction Times and Directs the Outcome of Differentiation

**DOI:** 10.3389/fimmu.2016.00006

**Published:** 2016-01-25

**Authors:** Nicholas van Panhuys

**Affiliations:** ^1^Division of Experimental Biology, Sidra Medical and Research Center, Doha, Qatar

**Keywords:** TCR, differentiation, T cell, signal strength, cytokine

## Abstract

The ability of CD4+ T cells to differentiate into effector subsets underpins their ability to shape the immune response and mediate host protection. During T cell receptor-induced activation of CD4+ T cells, both the quality and quantity of specific activatory peptide/MHC ligands have been shown to control the polarization of naive CD4+ T cells in addition to co-stimulatory and cytokine-based signals. Recently, advances in two-­photon microscopy and tetramer-based cell tracking methods have allowed investigators to greatly extend the study of the role of TCR signaling in effector differentiation under *in vivo* conditions. In this review, we consider data from recent *in vivo* studies analyzing the role of TCR signal strength in controlling the outcome of CD4+ T cell differentiation and discuss the role of TCR in controlling the critical nature of CD4+ T cell interactions with dendritic cells during activation. We further propose a model whereby TCR signal strength controls the temporal aspects of T–DC interactions and the implications for this in mediating the downstream signaling events, which influence the transcriptional and epigenetic regulation of effector differentiation.

## Introduction

CD4+ T cells constitute a key population in the adaptive arm of the immune response, due to their ability to differentiate into various distinct effector populations such as Th1, Th2, Th17, T follicular helper (Tfh) cells, and induced regulatory (iTreg) T cells, which help to orchestrate host defense against various classes of pathogens (1, 2). The differentiation of naive CD4+ T cells into polarized effectors has been studied extensively, and two alternative mechanisms of cell fate control have been described. The predominant view espoused by the field is that following the activation of a CD4+ T cell by an antigen-presenting cell (APC) displaying cognate peptide in the context of MHCII CD4+ T cells differentiate into highly specialized subsets according to the cytokine milieu present during activation and division (1, 3, 4). However, in addition to this qualitative/cytokine-based model of differentiation, the literature also contains clear evidence for a quantitative mechanism that controls cell fate decisions. Quantitative models indicate that upon activation, CD4+ T cells are able to determine the strength of TCR signaling in terms of both the antigen load and quality of peptide presented, and in combination with co-stimulatory molecules, CD4+ T cells perform a cellular calculus to determine activatory signal strength. T-helper cells then use this qualitative signaling information to determine which program of differentiation to engage in (5).

This review aims to discuss the evidence that supports the strength of signal-based mechanisms for inducing differentiation in parallel with qualitative mechanisms and argues that TCR signaling dynamics include a temporal component that regulates the interactions between APC and CD4+ T cells, which explains much of the observed qualitative and quantitative phenomena previously described.

## Qualitative Mechanisms of T-Helper Cell Differentiation

Cytokine-based models indicate that CD4+ T cell differentiation is controlled by a qualitative signaling mechanism in which specific combinations of cytokines produced mainly by myeloid APCs (and also by non-hematopoietic tissue elements) induce a specific pathway of differentiation following antigen-dependent activation of CD4+ T cells. Following the activation of APC by pathogen-associated molecular patterns (PAMPs), APCs begin to upregulate the levels of both MHCII and co-activation molecules allowing for the activation of CD4+ T cells in an antigen-specific manner. PAMPs additionally induce the production of specific cytokines by activated APCs, which have been shown to induce the differentiation of specialized T-helper populations (6). Cytokines have been shown to directly induce the differentiation of T-helper subsets through binding to specific receptors on the surface of CD4+ T cells, the activation of specific patterns of JAKs and STATs, which then translocate to the nucleus, and the upregulation of master transcription factors that are essential for the determination and maintenance of cell lineage and controlling downstream effector functions (1, 7). Th1 cells are induced in response to infection with bacterial or viral pathogens and are also a hallmark of autoimmune disease. Th1 differentiation can be induced following exposure to IFNγ (8) or IL-12 (9) during activation and is characterized by the expression of the master regulator of transcription Tbet (10) and production of the effector cytokine IFNγ. Th2 differentiation occurs following infection with helminthic parasites and is also responsible for much of the pathology associated with asthmatic and allergic responses. Th2 cells are characterized by the upregulation of GATA3 (11) following differentiation and the ability to produce IL-4, -5, and/or -13 upon re-stimulation. While Th2 differentiation has classically been believed to be driven by IL-4 (12), several studies have indicated that IL-4 is not required for Th2 differentiation under *in vivo* conditions (13, 14). Additionally, cytokines such as IL-25 and IL-33 and TSLP have also been implicated to be involved in Th2 differentiation; however, these factors were not found to be required for the induction of differentiation (15). Thus, it has proved difficult to determine a specific qualitative cytokine signal responsible for Th2 differentiation under *in vivo* conditions, and it has been suggested that Th2 induction may occur through a default or endogenous pathway (16). Th17 cells are believed to play a role in defending against fungal pathogens and have also been found to play a significant role in several autoimmune diseases. The Th17 lineage is characterized by the expression of the transcription factor RORγt and by the expression of the cytokines IL-17, -21, and/or -22 (17). Tfh cells perform a specialized function in that after differentiation, they localize to the B cell follicle and are critical for providing B cell help, by inducing B cell affinity maturation, differentiation, and promoting the class switching of B cell immunoglobulin isotype expression. They are generally characterized by IL-21 expression along with the transcriptional repressor Bcl-6 and the surface receptors CXCR5 and PD-1 (2). iTreg cells are functionally distinct from other groups of T-helper cells as they play a key role in the immune response by dampening down excessive CD4+ T cell activation and controlling the extent of inflammation. While iTreg cells are characterized by the expression of the transcription factor Foxp3 and production of regulatory cytokines such as TGFβ and Il-10, these factors are also present in naturally occurring or thymically derived Tregs (nTreg), making it problematic to distinguish these two subsets under *in vivo* conditions (18).

These studies form a large body of compelling evidence indicating that the cytokine milieu represents a highly deterministic cellular cue during the polarization process. However, it should be noted that cell fate decisions occur due to a number of factors, and as the majority of qualitative/cytokine-based studies have been conducted at single antigen concentrations *in vitro* or *in vivo*, the quantitative/TCR signal strength-based component is generally not considered giving the appearance that cytokines dominate. However, in practice, this conclusion requires a more explicit analysis to assess the relationship and possible hierarchical and temporal dominance of qualitative vs. quantitative aspects of control.

## TCR Signal Strength (Antigenic Quantity/Quality) Directs CD4+ T Cell Differentiation

**Figure 1:**
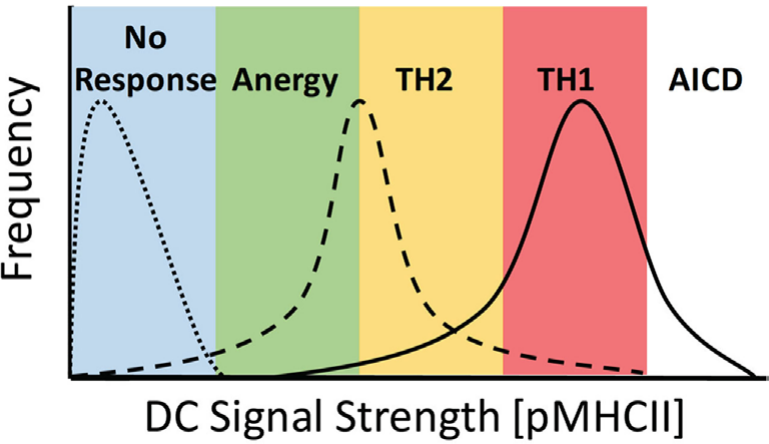
**Ability of DCs to stimulate TCR signaling controls the activation potential of CD4+ T cells**. DC priming of CD4+ T cells is dependent on the overall signal strength imparted by specific DC, where TCR signal strength = [peptide] × *K*_a_ of pMHCII. DC signaling capacity must be fitted to a distribution profile at the population level due to multiple factors, including expression levels of MHCII and co-stimulatory molecules, peptide loading efficiency, DC morphology, and availability of sites for interaction in a complex 3D environment. Here, DC lacking cognate peptide fail to stimulate a response from T cells (dotted line), whereas at low levels of signal strength, DC induce a complex response inducing anergy and Th2 and Th1 differentiation, with a predominant skewing toward TH1 differentiation (dashed line). When strong signals are present, the response is skewed toward TH1 differentiation with both Th2 differentiation and activation-induced cell death (AICD) also occurring (solid line).

The most widely characterized example of TCR signal strength directly contributing to the alternate differentiation of CD4+ T cells has been studied looking at the comparative differences in Th1 vs. Th2 induction. It has long been established that under *in vitro* conditions, a strong TCR signal leads to a predominance of Th1 differentiation, whereas weak TCR signaling leads to Th2 differentiation (19, 20). Early studies using an altered peptide ligand (APL) model system compared stimulation of TCR transgenic (Tg) CD4+ T cells with either the native Moth Cytochrome C (MCC) peptide or the K99 peptide, which has a significantly weaker affinity for the Tg TCR. When APCs were loaded with an equivalent quantity of antigen, those stimulated with the higher affinity MCC peptide displayed a striking tendency to differentiate into Th1 cells, whereas those stimulated with the weaker K99 peptide differentiated to the Th2 phenotype (19, 21–23). The capacity of APCs to induce TCR signaling can be modulated by both the affinity of the peptide:MHCII (pMHCII) complex for a specific TCR and the quantity of pMHCII present on the surface of the APC. Parallel studies examining the effects of peptide load on the differentiation CD4+ T cells revealed that stimulation with APCs loaded with a high dose of peptide led to the induction of Th1 cells, whereas stimulation with a low dose of peptide resulted in the differentiation of Th2 cells (20, 24). Together, these results show that altering the initial TCR stimulus received by naive CD4+ T cells, by either pMHC affinity or density, leads to a divergent outcome in terms of the end point of differentiation, with strong TCR signaling leading to Th1 differentiation and weak TCR signaling leading to Th2 differentiation (Figure 1).

Recent studies utilizing two-photon intravital microscopy and tetramer-based cell tracking methods have allowed investigators to greatly extend the study of the role of TCR signaling in effector differentiation under *in vivo* conditions. While the earlier studies discussed above imply that modulation of antigenic quality or quantity leads to similar outcomes at comparable levels of TCR stimulation, studies into the *in vivo* requirement for Treg generation by Gottschalk et al. (25, 26) indicate that TCR is able to differentiate between the two and that alteration of either of these components can affect the outcome of differentiation and/or survival, thus modulating the outcome of the immune response. Here, the induction of iTreg cell differentiation was assessed under *in vivo* conditions following the i.v. injection of MCC peptide or an APL to induce the activation of 5C.C7 TCR Tg CD4+ T cells. By adjusting the dose of MCC or APL peptide injected to normalize for peptide potency, comparable levels of proliferation and Foxp3 expressing iTreg cells were induced. However, those iTreg cells induced following activation with low-affinity ligands were unable to persist *in vivo*, illustrating the ability of TCR to distinguish between the quality and quantity of cognate peptide being presented under *in vivo* conditions (25). It was additionally determined that while large quantities of a weak agonist peptide and small quantities of a strong agonist peptide can provide a compensatory outcome in terms of *in vivo* proliferation, the stimulation of CD4+ T cells under these conditions led to divergent outcomes in terms of the gene expression profile-induced downstream. Further, intravital microscopy revealed that more stable contacts between CD4+ T cells and APC were observed in the presence of a strong agonist peptide in comparison to a weak agonist peptide, irrespective of the dose used (26), indicating antigen quality-dependent differences in T–DC interaction times may contribute to the observed divergence in gene expression profiles.

The differentiation of CD4+ T cells is influenced by TCR-mediated, co-stimulatory molecule and cytokine-induced signaling, all of which are modulated following APC activation by PAMPS or adjuvant-associated molecular patterns. The relationship between these factors in determining the outcome of differentiation is highly relevant. We recently addressed this issue by conducting a study utilizing the intravital imaging of CD4+ T cells interactions with DCs following their exposure to Th1- or Th2-polarizing adjuvants (27). Using Ca^2+^ flux imaging, these studies revealed a close correlation between the strength of TCR-induced signaling and the length of interaction, with Th1 differentiation being induced following increased TCR-associated signaling and longer lengths of interaction time. Under these conditions, antigen concentration was found to dominate over adjuvant in controlling differentiation. At a fixed antigen concentration, adjuvants inducing Th1 differentiation operated by increasing the co-stimulatory molecule expression on stimulatory DCs, which has previously been shown to potentiate TCR-associated signaling (28) and induce tighter T–DC interactions (29). However, by titrating the level of peptide used, we found that the concentration of antigen dominates the outcome of the response, such that, at high doses of antigen, CD4+ T cells primed with DC activated with Th2-polarizing adjuvants developed into Th1 cells and at low doses of antigen, Th2 differentiation predominated even after priming with DC activated in the presence of Th1-polarizing adjuvants. These responses showed a direct connection between alterations in Ca^2+^ signaling and interaction times, indicating that adjustments to TCR signal strength that affect interaction duration and signal accumulation can reverse the fate outcome, irrespective of the adjuvant used to treat the DC. These data imply that qualitative control of differentiation by cytokines reflects a secondary step in a developmental system in which the strength of initial T cell signaling regulates the capacity of the cells to respond to these mediators.

The majority of studies into the effects of TCR signal strength on CD4+ differentiation have relied upon the use of TCR Tg model systems; however, in order to more closely model responses occurring under physiological conditions, the application of tetramer-based technology has proved highly illuminating. During a polyclonal immune response, CD4+ T cells of various affinities for a given pMHCII are present in the immune repertoire; thus, it could be hypothesized from previous findings that those polyclonal cells with a high affinity for a specific pMHCII would preferentially differentiate into Th1 cells, whereas those with a lower affinity would differentiate into Th2 cells. To assess this possibility, Milner et al. (30) employed a tetramer-based staining system to deplete high-affinity CD4+ T cells and found that in the absence of such cells, stimulation of a polyclonal population with a specific pMHCII combination led to the preferential induction of a Th2 population, which was abrogated in the presence of higher affinity cells. These findings have been significantly extended by the use of an *in vivo* limiting-dilution system, which assessed the role of clonal differences in TCR signaling in response to a specific *Listeria monocytogenes* (Lm) peptide (31). Here, it was determined that single polyclonal cells responding to the Lm peptide displayed widely divergent abilities to differentiate into Th1, Tfh, and germinal center (GC) Tfh cells. However, when averaged together, these populations gave a characteristic pattern of differentiation, indicating that the diverse behaviors of individual naive CD4+ T cells with differing affinities for a specific peptide are able to account for the consistent pattern of response, which is found in a responding population of CD4+ T cells under *in vivo* conditions (31). Additionally, this study also indicated that the differentiation of Th1, Tfh, and GC-Tfh cells during Lm infection was dose dependent, with low-dose Lm infections leading to the predominance of Th1 and Tfh cells and high-dose infections leading to a corresponding decrease in the proportion of Th1 cells and an increase in the proportion of Tfh cells, especially GC-Tfh cells. An *in vivo* comparison of CD4+ T cells responding to whole PCC protein immunization further showed that precursor T cells with a higher affinity for pMHCII preferentially differentiated into Tfh cells and that LN-resident Tfh cells displayed a restricted TCR repertoire and displayed stronger pMHCII binding, (32) reinforcing the idea that Tfh cells are inducible in response to strong TCR signaling. Mechanistically, it has been shown that the generation of Tfh cells requires strong/sustained TCR signals through the analysis of the requirement for ITAM multiplicity in TCR signaling during Tfh cell generation, where it was determined that the presence of mutant ITAMs lacking their p-Tyr residues decreased proximal signaling downstream from the TCR and led to a concomitant decrease in Tfh cell generation (33). *In vivo* studies using 2P imaging to study the dynamic components of both B–T (34) and DC–T (35) interactions during Tfh cell responses have demonstrated that Tfh cells most frequently interact with APCs presenting strong pMHCII signaling complements and that prolonged Ag presentation was required (≥72 h) in order to most efficiently induce Tfh cell differentiation (36, 37). Together, these data indicate that very strong TCR stimuli are associated with the continued interaction of DC and CD4+ T cells for extended periods of time and that these ongoing interactions are required for Tfh cell differentiation.

Th17 differentiation has also been observed to occur in a dose-dependent manner; initial studies indicated that *in vitro* Th17 differentiation occurs in response to stimulation with a strong pMHCII stimulus, in conjunction with the presence of exogenous TGFβ and IL-6. Here, it was found that high doses of antigen were able to upregulate CD40L, and in combination with specific adjuvants, this increased DC IL-6 production leading to the differentiation of Th17 cells under *in vitro* and *in vivo* conditions (38). Interestingly, in the presence of IL-6 and TGFβ, the ability of naive CD4+ T cells to differentiate into Th1 or Th2 cells was abrogated, but Th17 differentiation was found to correlate with the strength of antigenic stimuli. CD4+ T cells deficient for Itk, a tyrosine kinase activated as part of the proximal response to TCR activation, have additionally been shown to have a decreased TCR-induced Ca^2+^ flux response (39) to antigen and have a deficiency in Th17 differentiation, even in the presence of IL-6 and TGFβ (40). Conversely, Itk-deficient cells exhibited increased Treg cell differentiation under Th17 conditions, where cells were found to be more sensitive to IL-2 signaling while exhibiting an activation profile similar to that observed in cells activated with a weak TCR stimulus (41). This observation indicates that Treg and Th17 differentiation may result from stimulation under a similar cytokine milieu, but their alternate induction is driven by TCR stimulation at opposite ends of the activation spectrum. Together, this literature clearly indicates that the predominant thesis describing CD4+ T cell differentiation by qualitative cytokine-based induction needs to be updated to better integrate the wealth of studies demonstrating a significant role for quantitative mechanisms in differentiation.

## TCR Signal Strength Influences Mechanisms of Qualitative Signaling During Differentiation

Naive CD4+ cells display an array of cytokine receptor molecules on their surfaces, such as IL-4R, IL-6R, IL-17R, IFNγR, and TGFβR, which are important for the induction of multiple differentiation programs. However, upon TCR activation, the expression of additional cytokine receptors such as the high-affinity IL-2R (CD25) (42) and the IL-12Rβ2 (43) is induced, and levels of existing cytokine receptors may be modulated according to signal strength (44). Interestingly, cytokines, such as IL-12 (45) and IFNγ (46, 47), associated with Th1 differentiation, have been shown to require the formation of mature immune synapses (IS) for their efficient delivery, with cytokine receptors localizing to the IS following formation of a mature IS, whereas IL-4 associated with Th1 differentiation (12), but not necessary for Th2 differentiation (14), is thought to act in a paracrine fashion, as does IL-2. Accordingly, these cytokines are not secreted in a directional fashion and their receptors do not display polarization even upon formation of a mature IS (47, 48).

However, responsiveness to IL-2 signaling has been shown to be regulated by TCR signal strength; activation by both weak and strong stimuli leads to an increased expression of IL-2R, but strong stimuli also cause an attenuated ability to signal via IL-2R through inhibition of phosphorylation of the transcription factor STAT5 (24), induced by the dose-dependent repression of Pten (41). The dose-dependent control of IL-2 signaling may therefore be a major factor controlling the Treg–Th17 differentiation axis. As where Th1 and Th2 differentiation is inhibited by TGFβ, Treg and Th17 differentiation predominates (38, 41, 49). However, while IL-2 is essential for Treg differentiation (18), IL-2 signaling inhibits Th17 differentiation (50); therefore, the negative feedback on STAT5 phosphorylation induced by strong TCR signaling allows for Th17 differentiation in the presence of IL-2, similar to that produced by naive T cells at early time points after activation. Further, Th17 differentiation has been shown to be dependent on the production of IL-6 by DC; thus, the potential exists that Th17 induction may require high levels of TCR stimuli in order to form a mature IS to allow for the directional secretion of IL-6. Though the directionality of IL-6 secretion at the T–DC interface has not been fully investigated, IL-6 is thought to be synaptically secreted in the central nervous system (51). The repression of IL-2R signaling may also play a significant role in the differentiation of the Tfh cell lineage. Upregulation of Bcl-6 is required for Tfh cell commitment, as opposed to the expression of Blimp-1, which is expressed by T effector lineages (52). Blimp-1 is upregulated in naive T cells upon activation and exposure to IL-2 (53) and causes the inhibition of Bcl-6 expression repressing Tfh cell differentiation in a STAT5-dependent manner (54). Additionally, the anti-apoptotic qualities of Bcl-6 may serve a protective function during Tfh cell differentiation (55) by allowing their stimulation by the very strong TCR signals over a long period of time that are required for Tfh cell differentiation, while avoiding activation-induced cell death (AICD).

In terms of Th1 differentiation, we recently showed that under both *in vitro* and *in vivo* conditions, TCR signal strength controlled downstream IL-12 receptor expression, with IL-12Rβ2 directly correlating with signal strength. Linking TCR signaling to the ability of CD4+ T cells to respond to cytokine signaling and establishing TCR-induced signaling play a dominant role in the signaling hierarchy (27). Together, these data indicate that CD4+ T cells display an ability to quantitate and integrate signals from the TCR and co-stimulatory molecules, which then regulates both the length of interaction and downstream checkpoints, such as the upregulation of cytokine receptors and their polarization to the IS, which is required for the cytokine-mediated control of effector differentiation. As the strength of signal received through TCR can alter the interaction time between T cells and DC, this indicates that it is an integral component in determining the outcome of differentiation.

## A Temporal Interaction-Based Model to Describe TCR-Driven Differentiation *In Vivo*

### *In Vivo* Modeling of T Cell Migration

T cells display a default scanning behavior under *in vivo* conditions possessing what could be thought of as an inbuilt “migrational momentum,” such that they will resist activatory “Stop” signals from an APC, unless a significant influence is exerted upon them. Multiple studies (26, 27, 34, 56) have shown a high degree of correlation between the length of time T cells interact with APCs and the outcome of differentiation, suggesting the need for a model of differentiation that takes into account the dynamic nature of T cell–DC interactions, the role of TCR in facilitating the nature of these interactions, and the pattern of resultant downstream signaling that regulates the outcome of differentiation.

T cells display a natural propensity for migration, with naive T cells estimated to recirculate though the secondary lymphoid organs in as little as a few hours as they search for cognate antigen (57, 58). Initial imaging studies (59, 60) assessing the behavior of naive CD4+ T cells in lymph node explant models characterized a short-lived contact duration between naive T cells and APCs of approximately 3 min. These Ag nonspecific interactions did not lead to T cell activation and were followed by a rapid dissociation and migration away from the APC, allowing the naive T cell to continue its scanning behavior. It was further noted that apart from brief pauses, naive T cells exist in a state of constant kinesis (61). The apparent random nature of *in vivo* migratory behavior observed in early studies led to the adoption of a random walk model, with T cells migrating as autonomous agents taking independent and stochastic paths through the secondary lymphoid tissue in search of APCs with cognate Ag (60, 61). However, more detailed studies examining the structure of secondary lymphoid organs characterized a complex matrix-like environment consisting of a network of interconnected follicular reticular cells along which T cells and APCs traffic (62). A particularly dense network of FRCs is associated near the HEVs in the paracortex, which enables the localization of tissue-derived DCs for the efficient presentation of Ag to immigrating T cells, and upon Ag administration, this site corresponds to areas of T cell clustering and early activation (63, 64). The expression of CCR7 by naive T cells and its requirement for migration into the lymphoid compartment is well documented (64, 65). However, the role it plays in guiding CD4+ T cells once inside the SLOs is less clear. FRCs in both humans and mice produce the CCR7 ligands CCL19 and CCL21 (66, 67) and likely guide the migration of naive CD4+ cells under steady-state conditions (67, 68). Upon inflammation, the potential role of chemokine gradients is yet to be fully determined, though activated dendritic cells have been observed to secrete CCL19 (69) and CCL21, and the expression can be downregulated by FRCs during an immune response (70). Ligation of CCR7 by CCL19 leads to receptor phosphorylation and internalization and a subsequent desensitization, potentially providing a mechanism for local CD4+ guidance, whereas CCL21 binding does not exhibit a reciprocal effect drawing naive CD4+ T cells toward activated DCs. The blockade (68) or disruption (71) of CCR7 ligand signaling has been shown to reduce the velocity of CD4+ migration, but failed to completely abrogate migration, indicating either the presence of additional as yet unidentified ligands or that CD4+ T cells exhibit a constitutively migrant behavior under *in vivo* conditions.

## The Initial Activatory Signal Provided by Kinetic Screening Allows Proofreading/Validation of Correct pMHC:TCR Matching

Upon interaction with APCs, it is vital for T cells to strictly discriminate between self and non-self ligands to avoid autoimmune disease, while at the same time maintain tolerance to nonpathogenic environmental antigens to avoid unnecessary pathology. *In vitro* studies have demonstrated that CD4+ T cells can be activated by less than ten cognate peptides presented by an APC in the presence of MHCII (72, 73); thus, T cell responses exhibit a very high degree of sensitivity. In order to scan the maximal number of APCs, non-productive interactions must be quickly terminated, such that in the absence of response to an agonist peptide, CD4+ T cell:APC interactions have been estimated to last between 1 and 5 min (59, 61, 74) and up to 5,000 T cells may contact and scan a single DC during an hour (74). The transition from TCR binding to signaling is highly specific, resulting in the ability to integrate TCR:pMHC interactions, which occur over a minimal range of *K*_d_ into a definitive digital on/off recognition signal (75). Thus, on the one hand, T cells must be able to rapidly and efficiently recognize their cognate Ag and, on the other, must display a rigorous intolerance to activation via the myriad of self-peptide and environmental antigens displayed by APCs.

In order to account for many of the observed details of TCR signaling and CD4+ activation, a kinetic thresholding with differential signaling feedback model of ligand discrimination has been suggested, (76) in particular, this mechanism of TCR signaling accounts for the speed, sensitivity, and specificity of signal discrimination. Kinetic thresholding models dictate that following pMHC:TCR interaction, proofreading kinases associated with the CD3 components of TCR phosphorylate the multiple tyrosine residues contained in the CD3 ITAM regions, allowing ZAP-70 association and progression of downstream signaling. Upon pMHC:TCR dissociation, the ITAMs are then dephosphorylated by constitutively active phosphatases, thereby attenuating the progression of signaling from the TCR. Thus, the duration of pMHC:TCR interaction sets the level of ITAM phosphorylation, which controls the lifetime of downstream signaling with the utilization of multiple proofreading kinases, allowing for the amplification of signaling in a nonlinear fashion (77–79). However, the initial kinetic proofreading models do not sufficiently account for all the characteristics of CD4+ activation; thus, in order to extend these models, an additional differential feedback step as identified by Stefanova et al. (80) was included. Here, Erk and SHP-1 were identified as key components of signal discrimination during the kinetic proofreading process. Following TCR engagement by both antagonistic and agonistic ligands, Lck is activated, resulting in its association and phosphorylation of both ZAP-70 and ITAMs, and Lck additionally phosphorylates the regulatory phosphatase SHP-1. In the case of TCR association by antagonistic or weak ligands, phosphorylation of SHP-1 allows binding to and inactivation of Lck and further enables a close association of SHP-1 with ZAP-70 and ITAMs, potentially resulting in the dephosphorylation of unprotected residues and cessation of signaling. However, following association with a strong agonist pMHC, the MAP kinase ERK is rapidly activated and phosphorylates Lck at Ser^59^, inhibiting the binding of SHP-1 and the subsequent inhibition of Lck kinase activity. Thus, ERK activation by strong agonists acts at a local level to protect Lck activity at TCR associated with agonist activation, causing the stabilization of ITAM phosphorylation and preventing desensitization through the subsequent binding of pSHP-1, which also accumulates following strong agonist binding. Under this model of TCR activation, signaling occurs in a binary fashion, with pSHP-1 inhibiting the activation unless threshold levels of agonist pMHC are encountered, whereupon, the system shifts to a fully activated state with full Erk phosphorylation, ZAP-70 activation, and phosphorylation of ITAMs.

Initial recognition of a pMHC:TCR match is amplified by Lck activation and phosphorylation of ZAP70, decreasing the activation threshold on neighboring TCR allowing for signal spreading by activation through interaction with lower affinity (self)pMHC:TCR interactions, which would be agonistic under steady-state conditions. It has been proposed that once activated ERK (76, 80) and/or Lck can act in a trans fashion, exerting effects on neighboring TCR to reduce the kinetic threshold of pMHC-TCR lifetime required for activation. Here, the transactivity of ERK and/or Lck may lead to a localized alteration in the balance of ITAM kinase vs. phosphatase activity, such that the speed of full ITAM phosphorylation occurs more rapidly than the dissociation of the pMHC:TCR complex, thereby causing TCR activation and recruitment of ZAP-70 in response to weak agonistic ligands such as self-peptides (81), resulting in a signal spreading mechanism and the formation of a local microcluster, which allows for the induction of TCR signaling from a small number of strong ligands. However, it is debatable whether this gives a better account for the stoichiometry of TCR downregulation than previously proposed models that envisage a serial engagement of pMHC by TCR with fast kinetics to account for the sustained signaling observed (82, 83). Additionally, signal spreading may provide a mechanism for the downstream calculation of TCR signal strength that cannot be accounted for by a binary mechanism of TCR activation.

## pMHC:TCR Signaling Induces Multiple Downstream Pathways With Distinct Effects on the Duration of Interactions and the Outcome of Differentiation

**Figure 2:**
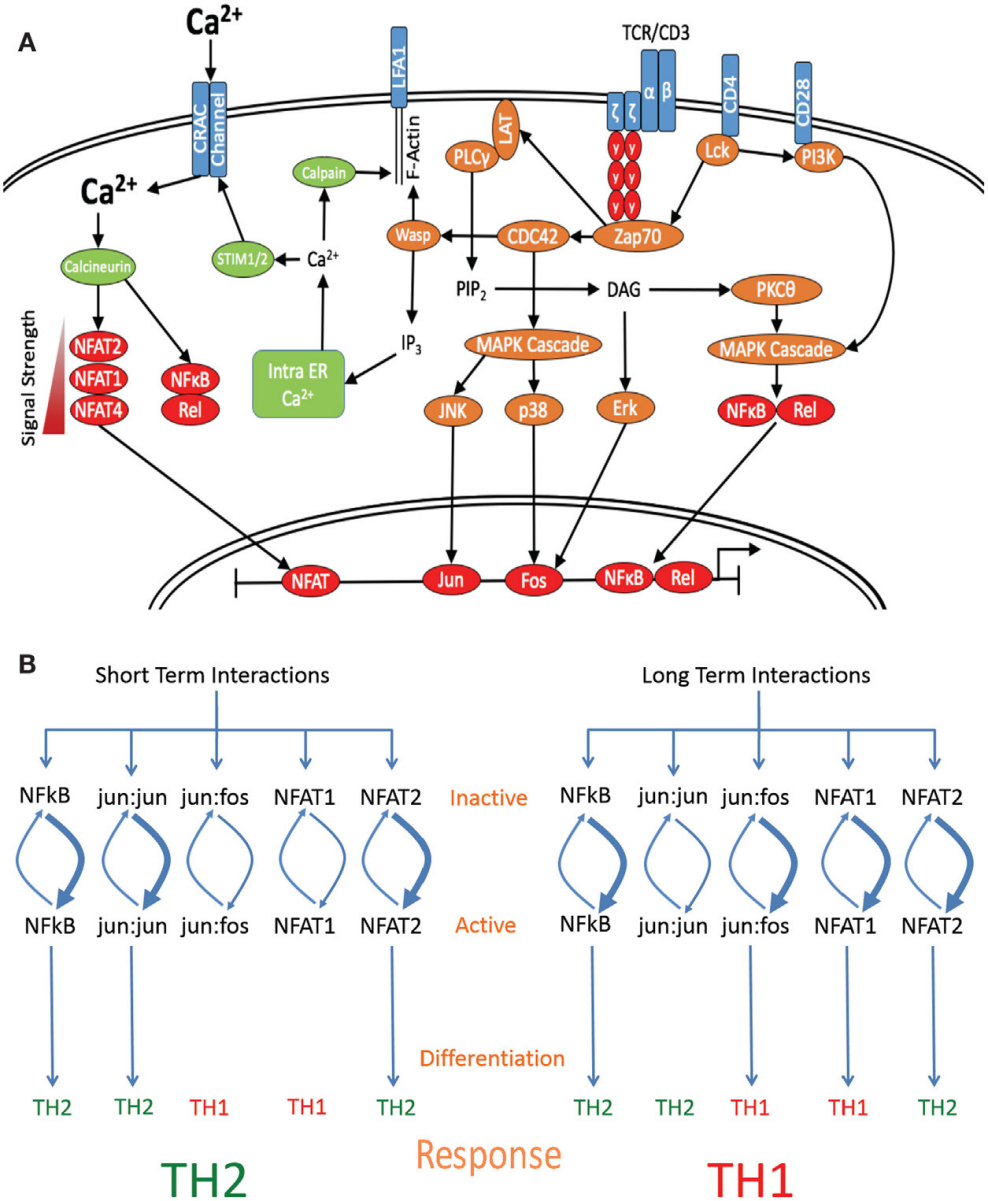
**Length of interaction alters the activation of specific transcription factors**. **(A)** Schematic diagram denoting the major signaling molecules involved in the T cell activation process upon stimulation of the TCR by cognate pMHCII, leading to the differential activation of downstream transcription factors based on signal strength and interaction times. **(B)** Short-term interactions induce a subset of default pathway of fast-acting transcription factors, which preferentially activate Th2-associated genes. Interaction for a longer period of time induces both default pathway-associated transcription factors and additionally allows for the induction of Th1-specific transcription factor activation, which are able to override the default Th2-inducing signal and skew the response toward the differentiation of a Th1 subset.

Each successful pMHC:TCR interaction induces at least two major distinct pathways; the first pathway leads to the induction of a default activation program consisting of proximal MAPK phosphorylation and the second results in an increase of intracellular Ca^2 +^ (Figure 2A). In lymphoid organs, the initial phase of T cell:APC interactions has been observed to comprise a number of brief serial interactions, with CD4+ T cell interactions lasting an average of 11–12 min (60) and CD8+ T cell interactions lasting 5–6 min (84). However, the average velocity of T cells decreases after contacting APCs loaded with cognate peptides during this first phase of interaction, and transient increases in intracellular calcium levels are observed (85). The sustained level of interaction necessary to form a mature IS does not generally occur until later in the response. However, following the kinaptic recognition (86) of cognate pMHC, T cells may receive a stimulatory signal sufficient to induce activation and maturation (87, 88). We propose that this indicates that in the absence of a sustained increase in calcium signaling sufficient to facilitate a halt in T cell migration required for the formation of a mature IS, what could be considered a default “on” pathway is sufficient to initiate TCR-driven activation of downstream transcription factors (89, 90). Thus, signaling from the TCR is able to directly turn naive T cells “on” and initiate maturation and differentiation by a primary default mechanism following the discrimination of a positive TCR signal. Second, where a sufficiently strong TCR signal is received, this may cause a halt in T cell migration by mediating a sustained increase in the intracellular calcium, thus allowing the formation of the IS and a prolonged T cell:APC interaction (91), as required for Th1 differentiation. The ability of T cells to discriminate between APCs loaded with different activatory stimuli allows T cells to perform a calculus when deciding which DC to form a synaptic relationship with (56) and indicates that T cells have specific mechanisms for determining the quantity and quality of signal they are receiving, which in turn imparts specific effector information.

While T cells are able to calculate the level of signal imparted to them by a specific APC, a mechanism for this has not yet been fully ascertained. However, it is known that naive T cells are able to form a stable long-lasting IS within minutes when forced to interact with lipid bilayers or APCs in culture, indicating that *de novo* protein synthesis is not a prerequisite for IS formation (92). *In vivo*, T cells have been observed to progress through an initial priming phase (phase I) of activation where APC encounters consist of brief serial contacts, before transitioning to phase II where long-lasting contacts with APCs are maintained (60, 93). However, this initial priming phase does not seem to be required for induction of full T cell activation as following *in vitro* activation T cells may proceed directly to phase II with the formation of an IS. It is possible that *in vivo*, the priming phase is used as a mechanism to calculate the level of pMHC being presented, leading to a decision whether to form an IS, while under *in vitro* conditions, the pMHC concentrations used to study IS formation are sufficient to bypass phase I. Alternatively, as the physiological signals that normally drive naive T cell migration are not present *in vitro*, this may lead to a reduction in cellular momentum and thus a decreased requirement for a brake-inducing signal. To account for the observed T cell behavior, such a stopping mechanism must be both quantitative for pMHC encountered and iterative with each APC interaction. One possibility for such an integrative function is the IP_3_-mediated release of intracellular Ca^2+^, whereby receptor signal spreading leads to an exponential increase in the quantity of TCR signaling complexes available to recruit and activate PLCγ causing a concomitant increase in IP_3_ production and signal strength-dependent release of Ca^2+^ from the ER. Huse et al. (94) observed that activation at decreased [pMHC] levels or blocking of CD4 binding increased the offset time for Ca^2+^ release, which suggests that a threshold level of IP_3_ is required for Ca^2+^ flux. Additionally, it has been demonstrated that *in vivo* activation of naive T cells with a low potency APL can induce upregulation of CD69 expression without inducing a sustained Ca^2+^ release, indicating the ability of T cells to be activated in the absence of sufficient TCR signaling to trigger Ca^2+^ release (85).

Once threshold levels of intra-ER Ca^2+^ are reached, store-operated calcium entry is then triggered via activation of STIM1 and STIM2, which sense Ca^2+^ release via low-affinity Ca^2+^-binding EF hand domains located in the ER lumen. Translocation of STIM molecules to the plasma membrane causes opening of CRAC channels, a sustained increase in intracellular Ca^2+^ levels, and initiation of a cellular brake response allowing for IS formation (32), as opposed to a brief kinaptic interaction. Initially, T cell momentum dictates the overall length of T cell:APC interaction, as Ca^2+^ levels must reach the threshold for CRAC channel activation within a predetermined period of time in order to induce a T cell braking response; otherwise, migration will continue. As in the absence of CRAC opening, the T cell fails to arrest, and the kinaptic interaction will be broken, with ER Ca^2+^ slowly returning to baseline, such that, ER Ca^2+^ levels may form an intracellular clock based on Ca^2+^ reuptake vs. [pMHC] stimulation.

Several studies (73, 85, 90, 95) have reported the correlation between the arrest of naive T cell migration and an increase in Ca^2+^ signaling following encounter with APC, although a mechanism for this decreased motility has not been completely described (86, 89, 96, 97). What is evident from this body of work is that the spike in intracellular Ca^2+^ levels following TCR-induced CRAC opening marks the transition from a migratory phase to a primary signaling phase, which may better account for the T cell ability to quantitate [pMHC]. It has been reported (98) that following TCR activation, WASp-deficient T cells displayed normal IS formation initially; however, after further migration, WASp-deficient T cells were unable to reform a stable IS in the presence of PKCθ. Conversely, PKCθ-deficient T cells formed hyper-stable IS and displayed impaired migratory ability. Sims et al. (98) suggest that PKCθ is required for the periodic breaking of IS symmetry, which allows for bursts of mobility during phase I of activation, with WASp playing an opposing role in re-establishing symmetry upon encounter with subsequent APCs. Thus, the interplay between PKCθ and WASp provides a mechanism for the quantitation of [pMHC]. Large increases in intracellular [Ca^2+^] are associated with a decrease in T cell motility, with Ca^2+^ flux signaling initiating the reorganization of f-actin function, from providing the mechanical force necessary for migration (99) to one whereby f-actin stabilizes the IS (89). F-actin clustering is additionally required to stabilize activated LFA-1 at the cell surface (96); thus, f-actin-stabilized LFA-1 persistence at the cell surface may represent a further downstream mechanism for determination of antigen signal strength and may link PKCθ/WASp activity to the transition between phase I/phase II T cell migratory behavior. Additionally, the ER/CRAC channel circuit in T cell displays capacitor-like behavior, with the ability to rapidly evoke a significant increase in the intracellular Ca^2+^ level followed by a period of heightened intracellular [Ca^2+^] as Ca^2+^ ions are removed by specific pumps. At this time, the IP_3_R is unresponsive to further signaling as bound Ca^2+^ on the cytosolic side of the ER inhibits IP_3_ binding (100). During this period, the TCR-associated activation of WASp may organize f-actin stabilization of LFA-1 (and other integrins) at the kinaptic interface in response to pMHC levels, whereas upon decrease of Ca^2+^ to sufficient levels, PKCθ activity induces a redirection of f-actin polarization away from the T cell–APC interface, destabilizing the connection and allowing the resumption of migration.

Studies have shown that LFA-1 is required for optimal *in vivo* priming (101) and that LFA-1 decreases the antigen dose required for Th1 differentiation (102). Thus, an increase in activated LFA-1 at the cell surface may serve as a molecular counter for previous TCR:pMHC interactions, whereby upon the cumulative activation of sufficient LFA-1, the rate of polymerization of f-actin at the T cell:APC interface is greater than the PKCθ-induced migration-associated f-actin polymerization, enabling maintenance of the kynapse long enough for the ER to refill and induce recurrent calcium spiking. In turn, inhibiting PKCθ-induced migration for a sufficient period of time allows for the formation of a mature IS. Mechanistically, this provides an explanation for previous data indicating that the transcription of Ca^2+^-associated gene targets is more efficiently activated by oscillating calcium levels rather than a sustained increase in Ca^2+^ levels (91, 103, 104).

## A Case Study in Temporally Regulated Differentiation: Th1 vs. Th2 Induction

### Induction of Sustained Signaling Through IS Formation Promotes a Program of Differentiation Favoring Th1 Development

It is now well established that the stimulation of naive CD4+ T cells with a strong agonist or high dose of peptide leads to the induction of Th1 differentiation, while low dose or weak APL stimulation leads to the induction of Th2 differentiation (19, 21, 22, 24, 105). However, a molecular mechanism to explain these phenomena has not yet been adequately provided. Much of the data generated on the induction of Th1 differentiation have indicated that the required signal may be imparted through the formation of a mature IS. Knockout studies have revealed that many of the components, which link TCR activation with IS formation such as the calcium signaling (106–108) and actin polarization pathways (109, 110), are also required for Th1 differentiation. Thus, it is possible that transcription factors, which are regulated proximally to TCR signaling, are regulated in a temporal fashion, whereby long-term activation is required for the optimal activation and function of Th1-associated transcription factors such as Jun Kinase (JNK) (22), Erk1/2 (22, 24), AP-1 (fos:jun) (22), NFAT-1, and NFAT-4 (21). Several possible mechanisms exist that may account for the requirement for long-term signaling; Th1-associated transcription factors may take longer time to be phosphorylated and translocate to the nucleus, as suggested by Brogdon et al. (21) where a weak TCR stimulus caused translocation of NFAT-2 to the nucleus and a strong stimulation led to the nuclear recruitment of both NFAT-2 and NFAT-1. Alternately, Th1-associated gene targets may be more inaccessible, requiring greater alterations to their methylation and acetylation states before transcription is possible. Third, Th1-associated transcription factors may be subject to a greater degree of regulation by inhibitory kinases or phosphatases after activation, leading to a diminished half-life, comparatively decreasing their ability to localize to the nucleus and hence their signaling capacity.

## Short-Lived Interactions Lead to the Initiation of a Default/Endogenous Activation Program Leading to Th2 Differentiation

In addition to low-dose/affinity peptide stimulation inducing Th2 differentiation, alterations to the TCR-associated signaling machinery can lead to the induction of Th2 differentiation. Inhibition of ZAP70 (111), a proximal TCR-associated kinase, which associates with phospho-ζ, was found to cause Th2 skewing, and a point mutation in the ZAP70 kinase domain has been described, which additionally induces Th2 skewing (112). As ZAP70 is thought to be required for full activation and efficient signaling, abrogation of its function is likely to lead to a decrease in the transduced signal strength and corresponding calcium flux, thus interfering with the ability of the cells to arrest and form a mature IS. Further, defects in LAT, which is phosphorylated by ZAP70 and is thought to act as a negative regulator of TCR signaling, have been described, whereby animals developed lymphoproliferative disorders with pronounced Th2 skewing (113). Here, it is likely that in the absence of sufficient negative regulation, weak signals are sufficient to activate cells but insufficient to induce full calcium flux and migratory arrest, thus leading to short-lived interactions and Th2 differentiation.

Th2-associated signaling has also been observed to be more dependent on activation of co-stimulatory pathways with defects in CD4 (23), CD28 (114), Lck (21), and PI3K (115), causing deficiencies in Th2 differentiation. As such, it is possible that as decreased overall signal strength is associated with Th2 induction, differentiation may be more highly reliant on co-stimulation in order to drive productive signaling, whereby signals that would not usually reach the threshold for activation or those that would lead to an anergic response can be modulated by effects of co-stimulatory receptor activation through Lck and its downstream effects on Zap70 induction, leading to a productive TCR engagement. Additionally, co-stimulatory signals lead to the preferential induction of the Th2-associated transcription factor NFκB1 (116, 117), through a pathway that requires PKCθ and IKKα signaling (118, 119). Thus, the reliance of Th2 induction on co-stimulation may play two roles whereby it serves to both ensure the transduction of weak TCR signaling in the presence of a strong danger signal and lead to the induction of Th2 differentiation via a default NFκB1-driven pathway in the absence of Th1 promoting transcription factors.

Other transcription factors known to affect differentiation include the AP1 binding elements fos:jun and jun:jun, which induce Th1 and Th2 differentiation, respectively (1, 22). While jun:jun homodimers are sufficient to bind DNA elements, jun:fos heterodimers are more energetically stable and thus have higher binding efficiency and target affinity, allowing binding to a wider range of targets (120, 121). However, the efficiency of dimer formation is temporally controlled through the regulation of p38 and Erk, which activate fos and JNK and then jun (120). p38, Erk, and JNK are cytosolic proteins that require phosphorylation by upstream kinases in order to migrate to the nucleus and are also subject to heavy regulation by phosphatases. Upon nuclear entry, this sets up a highly dynamic situation whereby variations in the phosphorylation: dephosphorylation rates of p38, Erk, and JNK lead to differential nuclear ratios of active fos and jun (122). The temporal model put forward here predicts that short-term signaling would lead to high levels of active jun and Th2 induction, whereas nuclear localization of p38 and Erk would occur later, increasing active fos levels and favoring jun:fos formation and Th1 induction. In support of this theory, it has been previously reported that Erk signaling is maximal at both high peptide doses (24) and where strong agonists are used (22), whereas low peptide doses and weak agonists induced low levels of Erk activation (24). Further, where weak agonists were used or Erk was inhibited, jun:jun homodimer formation was found to far exceed that of fos:jun formation and was also associated with early production of IL-4 (22). This indicates that strong TCR signals, which also induce long-term interactions, are required for full Erk activation, fos:jun formation, and Th1 induction, where as weak signals induce short-term interactions and jun:jun formation, favoring Th2 differentiation (Figure 2B).

## Summary

Here, a temporally based mechanism of differentiation is suggested that accounts for many of the observed aspects of strength of signal-induced differentiation and the downstream signaling pathways associated with Th1 and Th2 differentiation. In this model, long-term interactions induce a program of Th1 differentiation and short-term interactions induce a program of Th2 differentiation. Integrating TCR signal strength through downstream signaling machinery such as calcium release allows for the influence of additional signaling systems such as co-stimulatory molecule activation and potentially cytokine signals to be combined to dictate the length of interaction between DC and CD4+ T cells and thus contributes to downstream effector differentiation (27). By varying the length of interaction time, cell fate is able to be controlled through utilizing the activation potentials of both fast-acting transcription factors such as NFAT2 and jun:jun and slow-acting transcription factors such as NFAT1 and fos:jun, with shorter interactions favoring Th2 induction and longer interactions Th1 induction. As these transcription factors are tightly regulated by both kinases and phosphatases, differentiation requires an interplay between the cycling of transcription factors between inactive/cytosolic and active/nuclear states, such that threshold concentrations of localized activated factors may be required in order to mediate the formation of epigenetic modifications and the switching on or off of the genes that control both differentiation state and effector function (3). As such, this level of control would allow for tightly regulated molecular signaling events to be transduced in a global fashion through modulation of multiple transcription factors by a temporal mechanism.

From a biological point of view, this temporal model helps explain several phenomena. As interaction time is relative to total signal strength, a model where marginal signaling such as engagement of only a few pMHC or use of very weak APL or ­activation in the absence of co-stimulation leads to the induction of an anergic program, and weak signaling induces Th2 ­differentiation and strong signaling causes Th1 induction. This provides a potential explanation for how tolerance to environmental antigens is induced, how the immune system detects a parasitic infection, and how environmental allergens induce Th2-associated allergic disease, whereas self-antigen recognition leads to Th1-associated autoimmune disease. Tolerance to common environmental antigens is generally induced as a normal response to the presentation of low levels of antigen in the absence of co-stimulation and thus induces a weak TCR response and an anergic program due to abrogated signaling and interactions (123). Nematode parasites on the other hand represent a ­significant pathogenic burden and secrete a large array of antigens as well as induce danger signals as they migrate through host tissue. Thus, in order to avoid host responses, parasites secrete molecules that downregulate the function of APCs and, while undergoing maturation within the host, turn over their antigenic profile, in some cases multiple times, during the course of their lifecycle. As such, parasites only secrete a specific antigen for a limited time, thus decreasing host recognition (124, 125). Further, the amount of specific antigen taken up by an individual DC is likely to be very small due to the inability of DC to phagocytize parasites, forcing reliance on endocytosis or pinocytosis for uptake of secreted Ag. Considering this, it is likely that as an evolutionary mechanism to combat nematode infection, the immune system has evolved to recognize parasites in terms of a moderate–to-low signal strength, which then allows for full activation via an endogenous pathway, in the absence of long-term signaling, which induces a program of differentiation culminating in a Th2 response. Similarly, when the immune system is stimulated by common environmental allergens, which are often capable of inducing danger signals (126), there may be a fine balance between inducing anergy and inducing a Th2 response, such that in the case of an initial encounter with an allergen in sufficient quantity or potency, a Th2 response may be induced, which when combined with chronic antigen exposure induces allergy. Conversely, autoimmune diseases tend to be predominantly Th1 or Th17 associated (127), which may be due to the thymic conditioning of the CD4+ repertoire through negative selection, such that CD4+ cells are generally non-reactive to self, except where a significant stimulus of sufficient quality is encountered (128). Such strong signaling may activate any cells remaining in the repertoire that are able to recognize self-antigen, and through recognition of a strong signal, a program of long-lasting interactions is induced leading to Th1or Th17 ­differentiation. During the induction of ­autoimmunity, strong TCR signaling may potentially occur due to the availability of a large amount of peptide via extensive presentation of self Ag or in the case of significant damage, where normally immune-privileged peptides are exposed in ­combination with a substantial danger signal. Evolutionarily, such signals are analogous to bacterial or viral infections, where a limited range of epitopes are presented during infection in high quantities along with the activation of specific PAMPs, leading to the delivery of high strength signals causing long-term interactions with DC and the induction of a Th1 response.

Incorporating a temporal component into our models of how CD4+ T cells interact with DC during Treg, Th17, and Tfh cell differentiation also allows for an explanation of some of the additional phenomena that have been reported to occur during differentiation, which are not fully explained by a purely qualitative model of cellular decision-making. A distinct parallel can be drawn between the differentiation of Th1–Th2 cells and Th17–Treg cells, with both Th1 and Th17 cells requiring a strong TCR signal and have been observed to interact for long periods of time with DC, whereas both Th2 and Treg cells require weak TCR signals and only interact for brief periods of time. Further, Th2 and Treg cells are able to differentiate either via endogenous signals or from cytokines that are secreted in a diffuse fashion, whereas Th1 and Th17 cells require additional cues that are delivered at the cellular synapse and require a strong and/or ongoing signal in order to regulate their ability to respond to the cytokines that direct their differentiation. Tfh cell differentiation has also been observed to have a temporal component with ongoing interactions being required for differentiation, and as such, it is appealing to hypothesize that their expression of Bcl-6 may allow for this ongoing interaction in a non-deleterious fashion, while they acquire additional interaction-based signals from DC in the form of factors such as ICOSL (52) and CD40L (38).

In summary, a temporal signaling model would explain how many of the observed alterations in TCR signaling modulate differentiation by linking TCR signal strength, co-stimulation, and cytokine inputs through a mechanism that allows for the integration of multiple signaling inputs into an overall measure of signal strength and outputs these as divergent programs of behavior during priming, which ultimately leads to alterations in cell fate and effector function.

## Author Contributions

The author confirms being the sole contributor of this work and approved it for publication.

## Conflict of Interest Statement

The author declares that the research was conducted in the absence of any commercial or financial relationships that could be construed as a potential conflict of interest.
